# Risk factors for breast cancer in postmenopausal Caucasian and Chinese-Canadian women

**DOI:** 10.1186/bcr2465

**Published:** 2010-01-06

**Authors:** Carolyn Y Tam, Lisa J Martin, Gregory Hislop, Anthony J Hanley, Salomon Minkin, Norman F Boyd

**Affiliations:** 1 Campbell Family Institute for Breast Cancer Research, Ontario Cancer Institute, 610 University Avenue, Toronto, Ontario M5G 2K9, Canada; 2 British Columbia Cancer Research Centre, 675 West 10th Avenue, Vancouver, British Columbia V5Z 1L3, Canada; 3 Department of Nutritional Sciences, University of Toronto, 150 College Street, Toronto, Ontario M5S 3E2, Canada

## Abstract

**Introduction:**

Striking differences exist between countries in the incidence of breast cancer. The causes of these differences are unknown, but because incidence rates change in migrants, they are thought to be due to lifestyle rather than genetic differences. The goal of this cross-sectional study was to examine breast cancer risk factors in populations with different risks for breast cancer.

**Methods:**

We compared breast cancer risk factors among three groups of postmenopausal Canadian women at substantially different risk of developing breast cancer - Caucasians (N = 413), Chinese women born in the West or who migrated to the West before age 21 (N = 216), and recent Chinese migrants (N = 421). Information on risk factors and dietary acculturation were collected by telephone interviews using questionnaires, and anthropometric measurements were taken at a home visit.

**Results:**

Compared to Caucasians, recent Chinese migrants weighed on average 14 kg less, were 6 cm shorter, had menarche a year later, were more often parous, less often had a family history of breast cancer or a benign breast biopsy, a higher Chinese dietary score, and a lower Western dietary score. For most of these variables, Western born Chinese and early Chinese migrants had values intermediate between those of Caucasians and recent Chinese migrants. We estimated five-year absolute risks for breast cancer using the Gail Model and found that risk estimates in Caucasians would be reduced by only 11% if they had the risk factor profile of recent Chinese migrants for the risk factors in the Gail Model.

**Conclusions:**

Our results suggest that in addition to the risk factors in the Gail Model, there likely are other factors that also contribute to the large difference in breast cancer risk between Canada and China.

## Introduction

The age-adjusted incidence of breast cancer varies greatly around the world, with higher rates in developed than in developing regions [1]. For the period of 1998 to 2002, rates were 81 per 100,000 in Canada, 41 per 100 000 in Hong Kong, 31 and 35 per 100 000 respectively in Guangzhou City and Shanghai, urban areas in Mainland China, and 15 per 100 000 in rural areas in Mainland China [1]. Differences in age-specific breast cancer incidence between countries are especially large after menopause [1].

The reasons for the international variation in incidence rates are unclear. However, rates in low-risk populations increase after migration to high-risk areas and in subsequent generations approximate those of the host population [2]. Compared to women born in Asia, Asian-Americans born in the West, whose grandparents were also born in the West, have a rate of breast cancer about six times higher [2]. Since these populations are genetically similar, acculturation, which occurs after migration and includes changes in environmental and lifestyle factors, may explain changes in the levels of risk factors and rates of disease [2].

In this study, we compared breast cancer risk factors among three groups of postmenopausal Canadian women - Caucasians, Chinese women born in the West or who migrated to the West before age 21, and recent Chinese migrants. Results from previous studies suggest that these three groups of women are at substantially different risk of developing breast cancer. Recent Asian migrants to the West had lower breast cancer risk compared to those who had lived in the West for a decade or longer [2]. Also, mortality from breast cancer was significantly lower in Chinese migrants compared to the Western host population [3]. Reasons for differences in breast cancer risk between these various populations have not been completely elucidated. Differences in exposure to risk factors between our study groups may contribute to differences in risk of breast cancer between them. Conversely, factors that do not differ between these groups are unlikely to explain differences in breast cancer risk.

## Materials and methods

### General method

This was a cross-sectional study in three groups of postmenopausal women aged 50 or more: white Caucasians, at high risk of breast cancer; Chinese women born in the West or migrated to the West early in life, at intermediate risk; and recent Chinese migrants to Canada, at low risk. Western born Chinese and early Chinese migrants share similar genetic background to recent Chinese migrants but are expected to have lifestyles that are more similar to Caucasians. We recruited subjects in Toronto and in Vancouver, collected data on risk factors and diet by telephone interviews, and gathered information about anthropometric variables and physical activity at home visits. Fasting blood samples and 24-hour urine samples were also collected. All interviews were conducted by trained female trilingual interviewers in the language of the subject's choice: English, Cantonese, or Mandarin. Recruitment was carried out through mammography screening centres because a major aim of the study was to compare mammographic density in these groups. Results related to mammographic density will be reported separately. Written informed consent was obtained from each subject. Ethical approval of the study was obtained from the University Health Network (Toronto), the Ontario Breast Screening Program, and the University of British Columbia. In this paper, we will focus on epidemiological data, anthropometric measurements, and dietary acculturation.

### Study population

#### Recruitment

In Toronto, subjects were recruited between January 2003 and November 2005 through mammography units of the Ontario Breast Screening Programme (OBSP) and OBSP affiliated sites. Subjects were approached on site at the clinics or were sent a letter, with an explanation of the goals and procedures of the study, followed by a phone call, during which their eligibility was determined.

In Vancouver, subjects were recruited between February 2003 and August 2005 through centres of the Screening Mammography Programme of British Columbia (SMPBC). All subjects were recruited by telephone.

Among women approached and eligible for the study, 66% of Caucasians, 87% of Western born Chinese and early Chinese migrants, and 76% of recent Chinese migrants provided consent. Caucasians and recent Chinese migrants were frequency-matched by age.

#### Subject selection

Women were eligible if they had a mammogram within six months of the date of recruitment, were aged 50 or more, and postmenopausal, and met the definitions of ethnicity given below. Menopausal status in subjects who had not had a hysterectomy was defined as at least 12 months of continuous amenorrhea. Subjects who had had a hysterectomy were classified as postmenopausal if they also had an oophorectomy, or were aged 50 years or more.

To be eligible for the study, Caucasians and recent Chinese migrants had to be willing to take part in the telephone interview on risk factors and diet, to provide a blood sample, and to participate in a home interview about physical activity and anthropometric variables. Because of the limited numbers of early Chinese migrants and Western born Chinese in the population, the selection criteria for these subjects were relaxed and required only willingness to take part in the telephone interview on risk factors. Providing biological samples and/or participation in the other interviews was optional. Twenty-four percent of the Western born Chinese and early Chinese migrants who provided consent completed all study components.

Subjects who had a history of breast cancer, breast surgery reduction or augmentation, had taken exogenous hormones (including hormone replacement therapy, Tamoxifen, Raloxifene, thyroid hormone, and oral contraceptives) in the six months preceding their mammograms, or were taking a medically prescribed diet were excluded.

#### Definitions of ethnicity

##### White Caucasian

Subjects were classified as white Caucasian if they stated their ethnic or racial origin as white and Caucasian, and they and their grandparents were born in one or more of the following countries: Canada, United States, Australia, New Zealand or Europe, defined here as Austria, Denmark, Finland, France, Italy, the Netherlands, Sweden, Switzerland, and the United Kingdom, all countries with cancer registries and an age-adjusted breast cancer incidence at least twice that of Hong Kong.

##### Recent Chinese migrant

Subjects were classified as recent Chinese migrants if they stated their ethnic or racial origin as Chinese, and they and their grandparents were born in Mainland China or Hong Kong, and they had been resident in a *Western *country for 10 years or less. In this context we defined as *Western *any country except south/east Asian countries: Cambodia, Japan, Korea, Macao, Malaysia, the Philippines, Singapore, Taiwan, Thailand, and Vietnam. China has six cancer registries contributing data to Cancer Incidence in Five Continents [1] and has low age-adjusted breast cancer incidence.

##### Early Chinese migrant

Subjects were classified as early Chinese migrants if they stated their ethnic or racial origin as Chinese, and they and their grandparents were born either in Mainland China or Hong Kong, and they moved to a *Western *country (as defined above in definition of recent Chinese migrants) before age 21.

##### Western born Chinese

Subjects were classified as Western born Chinese if they stated their ethnic or racial origin as Chinese, they were born in a Western country (as defined above in definition of Caucasians), and had not lived in a south/east Asian country (as defined above) for more than 10 years.

### Data collection

#### Epidemiological data

Information about reproductive and menstrual history, hormone use, smoking and alcohol use, breast examination, radiation exposure, and family history of breast cancer were collected by telephone using a standardized questionnaire. The length of this telephone interview was approximately 30 minutes, and did not differ by the groups studied.

Complete data were available for all information collected except for age at menarche, exposure to x rays in the chest area, and first degree family history of breast cancer (some of our subjects were adopted). Percentages of missing values for these variables are as follows: age at menarche (0% in Caucasians, 1.4% in Western born Chinese and early Chinese migrants, and 0.5% in recent Chinese migrants); exposure to x rays in the chest area (1.7% in Caucasians, 2.8% in Western born Chinese and early Chinese migrants, and 0% in recent Chinese migrants); and first degree family history of breast cancer (5.3% in Caucasians, 9.7% in Western born Chinese and early Chinese migrants, and 9.0% in recent Chinese migrants).

#### Anthropometric measures

The subjects were weighed to 0.1 kg on a balance scale and measured for standing height and sitting height to 0.5 cm using a stadiometer (Seca 222) at a home visit. Leg length was determined by subtracting sitting height from standing height. Body mass index (BMI) was calculated as weight (kg) divided by height squared (m^2^). Hip and waist circumferences were measured to 0.1 cm with a tape measure and skin-fold thickness in the subscapular, suprailiac and triceps areas was measured to 0.2 mm using Lange calipers. Except for standing height and weight, two measurements were taken for all anthropometric variables and the mean of the two measures was used in data analysis. The research technicians who made these measurements were trained and certified by the Department of Athletics and Recreation, University of Toronto.

#### Dietary acculturation

Dietary acculturation was assessed by a questionnaire developed from a study of acculturation in Chinese-Americans and Canadians [4]. Items in the questionnaire were divided into two scales: a five-item Chinese Dietary Acculturation Scale and a 10-item Western Dietary Acculturation Scale, measuring Chinese and Western eating behaviour respectively.

The items on the Chinese Dietary Acculturation Scale includes: eating tofu, eating traditionally preserved vegetables, balancing yin/yang foods, avoiding cold foods and drinks, and eating a Chinese-style breakfast such as consuming congee and dumplings. The concept of yin/yang food balance, which is nutritional balance from a Chinese medicine viewpoint, was explained to any subjects who were unfamiliar with it. It involves balancing consumption of yin foods which tend to be cooling and moistening for the body (for example, watermelon), with yang foods which tend to be heating and drying (for example, deep fried foods).

The items on the Western Dietary Acculturation Scale includes: eating breads, rolls, or bagels, drinking carbonated beverages, eating ground beef or hamburgers, eating pizza or spaghetti with tomato sauce, eating any kind of cheese, drinking milk or eating milk products, eating cakes, pies, or cookies for dessert, eating packaged or prepared such as TV dinners, eating at a Western fast food restaurant, and eating (any types of foods) between meals.

The subjects were asked about their consumption of each item in the past month. A *yes *response, which includes eating the food even only once a month, was given a score of 1 and *no *response a score of 0 for each item. The Chinese and Western dietary acculturation scores were calculated as the mean of the response scores for each scale.

### Statistical methods

All analyses were conducted using the SAS statistical software package version 9 (SAS Institute Inc, Cary, NC, USA). Data were inspected for normality and transformations were made when necessary. Details of the transformations used are given in the footnotes of tables. Because of the small numbers of Western born Chinese and early Chinese migrants, we combined these two groups in the analyses. These two groups were not significantly different for most of the risk factors. Any significant or noteworthy differences between them will be discussed in the text. First, the characteristics of the groups were compared using analysis of variance (ANOVA) for continuous variables and chi-square tests for categorical variables. Next, overall and pairwise comparisons of risk factors among the study groups were performed using analysis of covariance (ANCOVA) for continuous variables and logistic regression for binary response variables. Age, education, and the interaction of age with ethnicity (age × ethnicity) were included in the models as potential confounders. For the comparison of anthropometric variables, study site was also included in the model to control for systematic differences between research staff in performing these measurements. Pairwise comparisons were adjusted for multiple comparisons using the Bonferroni method. For the comparison between recent Chinese migrants and postmenopausal Chinese women living in urban China, t-tests were carried out for numerical variables, and chi-square significance test for categorical variables. All *P *values were based on two-tailed tests and *P *< 0.05 was considered to be significant.

We used the Gail Model [5,6], a mathematical model for estimating individual breast cancer risk, to estimate five-year absolute risk. The risk factors in this model include age, age at menarche, age at first live birth, number of previous biopsies, presence of atypical hyperplasia on biopsy, and number of first-degree relatives with breast cancer. The Gail Model was developed based on data from Caucasians, and has not been validated for and therefore may not be applicable to Asian women. It has been shown to have modest discriminatory accuracy at the individual level, but to accurately predict breast cancer incidence in groups of women (that is, have good calibration) [7]. We estimated five-year absolute risk of breast cancer by applying the model to Caucasians with three different risk factor profiles: (1) risk factor profile of Caucasians, (2) risk factor profile of Western born Chinese and early Chinese migrants, (3) risk factor profile of recent Chinese migrants. Our goal was to assess the potential significance of the risk factors in the Gail Model in explaining the differences in breast cancer risk between our study groups. For the purpose of this study, a risk model with good calibration is sufficient.

## Results

### Characteristics of subjects

Table 1 shows selected characteristics of the subjects by group. Among 1050 subjects, 413 (39%) were Caucasians, 216 (21%) were Western born Chinese or early Chinese migrants (63 Western born Chinese and 153 early Chinese migrants), and 421 (40%) were recent Chinese migrants. Among Caucasians, 271 (66%) were from Toronto and 142 (34%) from Vancouver; among Western born Chinese and early Chinese migrants, 76 (35%) were from Toronto and 140 (65%) from Vancouver; and among recent Chinese migrants, 287 (68%) were from Toronto and 134 (32%) from Vancouver.

**Table 1:** Selected characteristics of subjects by study group^a^

	Mean (SD)						
							
		Chinese born in the West or migrated to the West before 21	Recent Chinese migrants	*P*			
	Caucasians			*P* ^b^	*P* ^c^	*P* ^d^	*P* ^e^
	(N = 413)	(N = 216)	(N = 421)				
Age (years)	61.5 (6.9)	58.5 (6.7)	60.3 (6.4)	<0.001	<0.001	0.03	0.004
Years lived in Western countries (years)	61.3 (6.9)	46.2 (12.7)	6.7 (2.9)	<0.001	<0.001	<0.001	<0.001
Education (% yes)							
Less than 8 years	1.0	3.3	15.0				
8 to 11 years	9.7	10.7	16.9				
High School	20.9	24.2	22.8	<0.001	0.04	<0.001	<0.001
Vocational/Technical School	7.5	10.2	9.0				
College	31.1	20.9	19.0				
Bachelor's Degree or higher	29.7	30.7	17.3				

^a ^*P *values were based on ANOVA for continuous variables and on chi-square tests for categorical variables. Pairwise comparisons were adjusted for multiple comparisons using the Bonferroni method. Continuous variables are expressed as means (SD). Categorical variables are expressed as percent.

^b ^*P *values for ethnicity effect

^c ^*P *values for comparing Caucasians and Chinese born in the West or migrated to the West before age 21

^d ^*P *values for comparing Caucasians and recent Chinese migrants

^e ^*P *values for comparing Chinese born in the West or migrated to the West before age 21 and recent Chinese migrants

The mean age of the study population was 60 years. Average length of residence in the West was 61 years for Caucasians, 46 years for Western born Chinese and early Chinese migrants, and 7 years for recent Chinese migrants. Recent Chinese migrants less often had a university education compared to the other two study groups.

### Reproductive risk factors

Table 2 shows the distribution of reproductive risk factors by group. Compared to Caucasians, recent Chinese migrants had later age at both menarche and first live birth, were more often parous, less often breastfed, had similar age at menopause, and less often reported to have ever used oral contraceptives or hormone replacement therapy. Western born Chinese and early Chinese migrants had values intermediate between the other two groups for most of the reproductive variables.

**Table 2:** Reproductive risk factors by study group^a^

	Adjusted Mean (Lower and Upper 95% Confidence Limits)						
					
				*P*			
				
	Caucasians	Chinese born in the West or migrated to the West before 21	Recent Chinese migrants	*P* ^b^	*P* ^c^	*P* ^d^	*P* ^e^
	(N = 413)	(N = 216)	(N = 421)				
Age at menarche (years)^f, g^	12.8 (12.6, 13.0)	13.0 (12.7, 13.2)	13.7 (13.6, 13.9)	0.01	0.64	<0.001	<0.001
Age at first live birth (years)^f, h^	24.3 (23.8, 24.7)	24.9 (24.3, 25.5)	25.5 (25.1, 25.9)	0.002	0.34	<0.001	0.22
Number of live births (%)							
0	14.5 (11.3, 18.5)	8.5 (5.2, 13.8)	4.5 (2.8, 7.2)	0.60	0.14	<0.001	0.19
1	9.9 (7.3, 13.2)	7.7 (4.6, 12.7)	18.0 (14.3, 22.4)	0.08	0.99	0.005	0.007
2	36.9 (32.3, 41.8)	40.1 (33.2, 47.4)	43.3 (38.5, 48.2)	0.14	0.99	0.22	0.99
3	21.6 (17.7, 26.1)	30.8 (24.7, 37.7)	19.6 (15.9, 37.7)	0.08	0.05	0.99	0.008
≥ 4	11.9 (8.9, 15.9)	5.3 (2.9, 9.6)	8.0 (5.7, 11.2)	0.33	0.05	0.24	0.71
Breastfeeding (% yes)	76.6 (71.6, 80.9)	63.4 (55.9, 70.2)	72.1 (66.9, 76.8)	<0.001	0.62	<0.001	0.14
Age at natural menopause (years)^f, i^	50.1 (49.7, 50.6)	50.6 (50.0, 51.3)	49.9 (49.5, 50.3)	0.79	0.53	0.99	0.10
Age at surgical menopause with both ovaries removed (years)^f, j^	44.0 (41.2, 46.9)	45.0 (41.3, 48.9)	45.0 (42.2, 48.0)	0.68	0.99	0.99	0.99
Ever used oral contraceptives (% yes)	85.8 (81.4, 89.3)	73.9 (67.3, 79.6)	40.7 (35.9, 45.7)	<0.001	0.002	<0.001	<0.001
Duration of oral contraceptive use (years)^f, k^	3.6 (3.0, 4.3)	2.5 (2.0, 3.2)	2.1 (1.7, 2.6)	0.14	0.03	<0.001	0.81
Ever used hormone replacement therapy (% yes)	47.4 (41.7, 53.1)	33.1 (26.6, 40.3)	28.1 (23.9, 32.6)	0.03	0.003	<0.001	0.65
Duration of hormone replacement therapy use (years)^f, l^	2.1 (1.6, 2.8)	1.7 (1.2, 2.6)	1.1 (0.8, 1.4)	0.01	0.99	0.001	0.17

^a ^*P *values were based on ANCOVA for continuous variables and logistic regression for binary variables, adjusting for age, education, and age × ethnicity. Pairwise comparisons were adjusted for multiple comparisons using the Bonferroni method. Variables are expressed as adjusted means.

^b ^*P *values for ethnicity effect.

^c ^*P *values for comparing Caucasians and Chinese born in the West or migrated to the West before age 21.

^d ^*P *values for comparing Caucasians and recent Chinese migrants.

^e ^*P *values for comparing Chinese born in the West or migrated to the West before age 21 and recent Chinese migrants.

^f ^Log transformed.

^g ^There were 3 missing values for Chinese born in the West or migrated to the West before age 21 and 2 missing values for recent Chinese migrants.

^h ^Sample size was 344 Caucasians, 191 Chinese born in the West or migrated to the West before age 21, and 397 recent Chinese migrants.

^i ^Sample size was 274 Caucasians, 159 Chinese born in the West or migrated to the West before age 21, and 345 recent Chinese migrants.

^j ^Sample size was 28 Caucasians, 13 Chinese born in the West or migrated to the West before 21, and 24 recent Chinese migrants.

^k ^Sample size of oral contraceptive uses was 313 Caucasians, 154 Chinese born in the West or migrated to the West before 21, and 169 for recent Chinese migrants.

^l ^Sample size of hormone replacement therapy users was 201 Caucasians, 68 Chinese born in the West or migrated to the West before 21, and 118 recent Chinese migrants.

### Anthropometric risk factors

Table 3 shows the distribution of anthropometric risk factors by group. Among 1,050 subjects, 382 Caucasians (93%), 53 Western born Chinese or early Chinese migrants (25%), and 357 recent Chinese migrants (85%) had measurements of body size made. Anthropometric measurement was not mandatory for Western born Chinese and early Chinese migrants. Within this group, participants and non-participants of anthropometric measurement were similar for all variables measured in this study, except for Western dietary score which was significantly higher in the participants (*P *= 0.02). We did not achieve full participation from all Caucasians and recent Chinese migrants because some of these subjects declined measurements after recruitment.

**Table 3:** Anthropometric risk factors by study group^a^

	Adjusted Mean (Lower and Upper 95% Confidence Limits)						
					
				*P*			
				
	Caucasians	Chinese born in the West or migrated to the West before 21	Recent Chinese migrants	*P* ^b^	*P* ^c^	*P* ^d^	*P* ^e^
	(N = 382)	(N = 53)	(N = 357)				
Standing height (cm)	162.1 (161.4, 162.8)	156.3 (154.6, 157.9)	155.8 (155.3, 156.4)	0.03	<0.001	<0.001	0.99
Weight (kg)	69.5 (68.1, 70.9)	57.7 (54.8, 60.8)	55.2 (54.2, 56.3)	<0.001	<0.001	<0.001	0.35
BMI (kg/m^2^)	26.4 (25.9, 26.9)	23.6 (22.5, 24.8)	22.7 (22.4, 23.1)	<0.001	<0.001	<0.001	0.40
Sitting height (cm)	85.0 (84.6, 85.4)	83.5 (82.6, 84.5)	83.3 (83.0, 83.7)	0.06	0.01	<0.001	0.99
Leg length (cm)	77.1 (76.6, 77.6)	72.7 (71.5, 73.9)	72.0 (72.0, 72.9)	0.14	<0.001	<0.001	0.99
Waist circumference (cm)	84.6 (83.3, 85.8)	77.4 (74.6, 80.4)	76.6 (75.6, 77.6)	<0.001	<0.001	<0.001	0.99
Hip circumference (cm)	104.5 (103.4, 105.6)	94.5 (92.1, 97.0)	93.4 (92.6, 94.3)	<0.001	<0.001	<0.001	0.99
Subscapular skinfold (mm)	30.0 (28.8, 31.3)	30.9 (27.8, 34.5)	27.3 (26.3, 28.4)	0.65	0.99	0.002	0.10
Suprailiac skinfold (mm)	30.9 (29.3, 32.5)	30.8 (27.0, 35.2)	27.7 (26.4, 29.0)	<0.001	0.99	0.003	0.38
Triceps skinfold (mm)	33.4 (32.3, 34.5)	32.9 (30.2, 35.8)	29.4 (28.5, 30.3)	0.04	0.99	<0.001	0.05

^a ^*P *values were based on ANCOVA, adjusting for age, education, age × ethnicity, and study site. Log transformation was used. Pairwise comparisons were adjusted for multiple comparisons using the Bonferroni method. Variables are expressed as adjusted means. Sample size for Chinese born in the West or migrated to the West before age 21 was much smaller than that for the other two groups because the selection criteria for these subjects were relaxed. Participation in the home interview for anthropometric measurements was optional. We did not get full participation from all Caucasians and recent Chinese migrants because some of these subjects declined measurements after recruitment.

^b ^*P *values for ethnicity effect

^c ^*P *values for comparing Caucasians and Chinese born in the West or migrated to the West before age 21

^d ^*P *values for comparing Caucasians and recent Chinese migrants

^e ^*P *values for comparing Chinese born in the West or migrated to the West before age 21 and recent Chinese migrants

Compared to Caucasians, recent Chinese migrants weighed less, were shorter in standing height, sitting height, and leg length, were smaller in both waist and hip circumferences, and were smaller in all skinfold thicknesses. Western born Chinese and early Chinese migrants were similar in skinfold thicknesses to Caucasians and were similar to recent Chinese migrants for the rest of the anthropometric variables.

### Other risk factors

Table 4 shows the distribution of other risk factors by group. Compared to recent Chinese migrants, Caucasians more frequently reported a first degree family history of breast cancer, having a benign breast biopsy, consuming alcohol at least once per week for six months or longer, or smoking at least one cigarette per day for three months or longer. Caucasians less often had a chest x-ray or an x-ray for tuberculosis detection than recent Chinese migrants. Western born Chinese and early Chinese migrants had values intermediate between the other two groups.

**Table 4:** Other risk factors by study group^a^

	Adjusted Mean (Lower and Upper 95% Confidence Limits) (% Yes)						
					
				*P*			
				
	Caucasians	Chinese born in the West or migrated to the West before 21	Recent Chinese migrants	*P* ^b^	*P* ^c^	*P* ^d^	*P* ^e^
	(N = 413)	(N = 216)	(N = 421)				
First degree relatives with breast cancer^*f*^	16.0 (12.0, 21.1)	14.7 (10.0, 21.0)	7.8 (5.5, 11.0)	0.87	0.99	0.003	0.04
Any benign breast biopsy	14.8 (11.5, 18.9)	8.7 (5.5, 8.2)	5.4 (3.5, 8.2)	0.04	0.10	<0.001	0.39
Consumption of alcohol at least once per week for six months or longer	60.6 (53.5, 67.2)	9.4 (6.0, 14.4)	2.3 (1.2, 4.2)	<0.001	<0.001	<0.001	<0.001
Consumption of alcohol at least once per week currently	50.9 (45.7, 56.0)	8.7 (5.6, 13.4)	1.4 (0.6, 3.3)	<0.001	<0.001	<0.001	<0.001
Smoked at least one cigarette per day for three months or longer	46.5 (39.1, 54.1)	12.4 (8.2, 18.2)	1.7 (0.8, 3.6)	<0.001	<0.001	<0.001	<0.001
Smoking at least one cigarette per day	11.0 (8.2, 14.6)	3.3 (1.5, 6.7)	0.5 (0.1, 1.9)	0.01	0.006	<0.001	0.05
Currently							
Any X-ray examinations that included the chest area^g^	73.7 (67.4, 79.1)	78.5 (71.1, 84.4)	97.4 (95.3, 98.5)	0.20	0.69	<0.001	<0.001
Any X-ray examination for tuberculosis^g^	58.3 (51.6, 64.7)	71.9 (64.3, 78.4)	96.4 (94.2, 97.9)	0.09	0.008	<0.001	<0.001

^a ^*P *values were based on logistic regression, adjusting for age, education, and age × ethnicity. Pairwise comparisons were adjusted for multiple comparisons using the Bonferroni method. Variables are expressed as adjusted means.

^b ^*P *values for ethnicity effect

^c ^*P *values for comparing Caucasians and Chinese born in the West or migrated to the West before age 21

^d ^*P *values for comparing Caucasians and recent Chinese migrants

^e ^*P *values for comparing Chinese born in the West or migrated to the West before age 21 and recent Chinese migrants

^f ^There were 22 missing values for Caucasians, 21 for Chinese born in the West or migrated to the West before age 21, and 38 for recent Chinese migrants.

^g ^There were seven missing values for Caucasians and six for Chinese born in the West or migrated to the West before age 21.

### Dietary acculturation

Table 5 shows the frequency of consumption of items on the Chinese and Western dietary acculturation scales and the distribution of dietary acculturation scores by group. Among 1,050 subjects, 379 Caucasians (92%), 152 Western born Chinese or early Chinese migrants (70%), and 374 recent Chinese migrants (89%) participated in dietary data collection. This was not a mandatory component for Western born Chinese and early Chinese migrants. Within this group, participants and non-participants of dietary data collection were similar for all variables measured in this study. We did not achieve full participation from all Caucasians and recent Chinese migrants because some of these subjects declined dietary data collection after recruitment.

**Table 5:** Dietary acculturation by study group

	Caucasians	Chinese born in the West or migrated to the West before 21	Recent Chinese migrants	*P* ^a^			
				*P* ^b^	*P* ^c^	*P* ^d^	*P* ^e^
	(N = 379)	(N = 152)	(N = 374)				
Chinese dietary practices (% yes)							
Eat tofu	20.8	89.5	95.7				
Eat traditionally preserved/pickled vegetables	40.1	49.3	56.7				
Balance yin/yang foods	7.1	23.7	33.2				
Avoid cold foods and drinks	11.9	29.0	44.7				
Eat a Chinese style breakfast (for example,. congee)	5.0	46.1	65.5				

Chinese Dietary Acculturation Score^f^	0.09 (0.08, 0.12)	0.42 (0.37, 0.47)	0.56 (0.53, 0.60)	<0.001	<0.001	<0.001	<0.001
[Adjusted Mean (95% Confidence Limits)]							

Western dietary practices (% yes)							
Eat bread, rolls, or bagels	96.6	94.1	94.9				
Drink carbonated beverages	63.9	50.0	27.0				
Eat ground beef or hamburgers	77.6	59.9	30.5				
Eat pizza or spaghetti with tomato sauce	89.2	79.6	52.7				
Eat any kind of cheese	97.1	80.3	49.7				
Drink milk or eat milk products	94.5	87.5	89.6				
Eat cakes, pies, or cookies for dessert	92.7	94.7	80.0				
Eat packaged or prepared foods (for example, TV dinners)	42.5	50.0	40.1				
Eat at Western fast food restaurants	40.9	50.0	33.2				
Eat between meals	81.3	90.1	80.9				
Western Dietary Acculturation Score^g^	0.80 (0.78, 0.81)	0.77 (0.74, 0.79)	0.64 (0.61, 0.66)	<0.001	0.10	<0.001	<0.001
[Adjusted Mean (95% Confidence Limits)]							

^a ^*P *values were based on ANCOVA, adjusting for age, education, and age × ethnicity. Pairwise comparisons were adjusted for multiple comparisons using the Bonferroni method.

^b ^*P *values for ethnicity effect

^c ^*P *values for comparing Caucasians and Chinese born in the West or migrated to the West before age 21

^d ^*P *values for comparing Caucasians and recent Chinese migrants

^e ^*P *values for comparing Chinese born in the West or migrated to the West before age 21 and recent Chinese migrants

^f ^Square-root transformed. The scores ranged from 0 to 1. A high score indicates positive association with Chinese dietary practices.

^g ^Cube transformed. The scores ranged from 0 to 1. A high score indicates positive association with Western dietary practices.

A higher score indicates positive association with Chinese or Western dietary practices. Recent Chinese migrants had a higher Chinese score and a lower Western score than Caucasians. Early Chinese migrants had Chinese and Western scores that were intermediate between those of Western born Chinese and recent Chinese migrants. Greater length of residence in Western countries was positively associated with a higher Western score in recent Chinese migrants (r = 0.15 and *P *= 0.003).

### Recent Chinese migrants by place of origin

Table 6 shows the distribution of risk factors in recent Chinese migrants by place of origin. Among recent Chinese migrants, 147 (34.9%) were from Mainland China, 133 (31.6%) were from Hong Kong, and 141 (33.5%) had lived in both Mainland China and Hong Kong. Compared to recent Chinese migrants from Hong Kong, those from Mainland China had lower exposure to all reproductive variables examined, were taller, less frequently had a first degree family history of breast cancer or a benign breast biopsy, and had a higher Chinese dietary acculturation score. Recent Chinese migrants who had lived in both Mainland China and Hong Kong had values intermediate between the other two groups of recent Chinese migrants for most of the risk factors.

**Table 6:** Selected breast cancer risk factors in recent Chinese migrants by place of origin^a^

	Adjusted Mean (Lower and Upper 95% Confidence Limits)						
	From	Had lived in both	From	*P*			
				
	Mainland	Mainland China	Hong Kong	*P* ^b^	*P* ^c^	*P* ^d^	*P* ^e^
	China	and Hong Kong					
	(N = 147)	(N = 141)	(N = 133)				
Age at menarche (years)^f, g^	14.3 (14.1, 14.6)	13.7 (13.4, 13.9)	13.0 (12.7, 13.3)	<0.001	0.004	<0.001	0.003
Age at first live birth (years)^f, h^	24.6 (24.0, 25.2)	25.7 (25.0, 26.3)	26.3 (25.6, 27.0)	0.003	0.07	0.003	0.47
Parity (% yes)	99.0 (95.5, 99.8)	97.4 (93.1, 99.0)	94.4 (87.9, 97.5)	0.06	0.86	0.12	0.53
Breastfeeding (% yes)	95.4 (89.9, 98.0)	66.7 (56.9, 75.1)	45.1 (34.1, 56.7)	<0.001	<0.001	<0.001	0.007
Ever used oral contraceptives (% yes)	29.5 (21.7, 38.8)	41.4 (32.8, 50.5)	49.6 (39.9, 59.4)	0.02	0.21	0.02	0.64
Ever used hormone therapy (% yes)^i^	12.8 (8.0, 19.7)	37.8 (30.0, 46.9)	35.6 (26.8, 45.4)	<0.001	<0.001	<0.001	0.99
Standing height (cm)^j^	156.5 (155.4, 157.5)	156.6 (155.6, 157.5)	154.5 (153.5, 155.6)	0.009	0.99	0.05	0.01
Weight (kg)^j^	55.9 (54.3, 57.6)	55.5 (54.0, 56.9)	53.8 (52.3, 55.4)	0.15	0.99	0.22	0.34
BMI (kg/m^2^)^j^	22.9 (22.3, 23.5)	22.6 (22.1, 23.2)	22.5 (22.0, 23.1)	0.77	0.99	0.99	0.99
First degree history of breast cancer (% yes)^k^	4.4 (1.9, 9.7)	5.1 (2.3, 10.8)	14.3 (8.3, 23.4)	0.02	0.99	0.06	0.07
Any benign breast biopsy (% yes)	2.9 (1.2, 7.0)	4.4 (1.9, 9.6)	7.8 (3.9, 14.9)	0.21	0.99	0.26	0.81
Chinese Dietary Acculturation Score^l, m^	0.64 (0.59, 0.68)	0.58 (0.54, 0.63)	0.50 (0.46, 0.54)	<0.001	0.34	<0.001	0.006
Western Dietary Acculturation Score^m, n^	0.62 (0.58, 0.66)	0.65 (0.61, 0.68)	0.65 (0.62, 0.68)	0.50	0.99	0.78	0.99

^a ^*P *values were based on ANCOVA for continuous variables and logistic regression for binary variables, adjusting for age, education, and years lived in the West. Pairwise comparisons were adjusted for multiple comparisons using the Bonferroni method. Variables are expressed as adjusted means.

^b ^*P *values for ethnicity effect

^c ^*P *values for comparing recent Chinese migrants from Mainland China and recent Chinese migrants who had lived in both Mainland China and Hong Kong

^d ^*P *values for recent Chinese migrants from Mainland China and recent Chinese migrants from Hong Kong

^e ^*P *values for comparing recent Chinese migrants who had lived in both Mainland China and Hong Kong and recent Chinese migrants from Hong Kong

^f ^Log transformed

^g ^There was 1 missing value for recent Chinese migrants from Mainland China and 1 missing value for recent Chinese migrants who had lived in both Mainland China and Hong Kong.

^h ^Sample size was 144 for recent Chinese migrants from Mainland China, 133 for recent Chinese migrants who had lived in both Mainland China and Hong Kong, and 120 for recent Chinese migrants from Hong Kong.

^i ^There were 2 missing values for recent Chinese migrants from Mainland China

^j ^There were 33 missing for recent Chinese migrants from Mainland China, 18 for recent Chinese migrants who had lived in both Mainland China and Hong Kong, and 13 for recent Chinese migrants from Hong Kong.

^k ^There were 5 missing values for recent Chinese migrants from Mainland China, 21 for recent Chinese migrants who had lived in both Mainland China and Hong Kong, and 12 for recent Chinese migrants from Hong Kong.

^l ^Square-root transformed. The scores ranged from 0 to 1. A high score indicates positive association with Chinese dietary practices.

^m ^There were 26 missing values for recent Chinese migrants from Mainland China, 12 for recent Chinese migrants who had lived in both Mainland China and Hong Kong, and 9 for recent Chinese migrants from Hong Kong.

^n ^Cube transformed. The scores ranged from 0 to 1. A high score indicates positive association with Western dietary practices.

### Selected breast cancer risk factors in recent Chinese migrants and postmenopausal Chinese women living in urban China

Table 7 shows selected breast cancer risk factors in recent Chinese migrants (99% of whom coming from urban China) and postmenopausal Chinese women living in Hong Kong [8,9] and Shanghai [10]. Compared to postmenopausal Chinese women living in Shanghai, recent Chinese migrants from Mainland China had significantly earlier age at menarche, later age at first live birth, later age at natural menopause, and lower BMI. Compared to postmenopausal Chinese women living in Hong Kong, recent Chinese migrants from Hong Kong attained menarche significantly earlier.

**Table 7:** Selected breast cancer risk factors in recent Chinese migrants and postmenopausal Chinese women living in urban China

	Mean (SD)			Mean (SD)		
	Recent Chinese Migrants	Postmenopausal		Recent Chinese Migrants	Postmenopausal	
	From Mainland China	Chinese Women	*P* ^a^	From Hong Kong	Chinese Women	*P* ^a^
	in This Study	Living in Shanghai^b^		in This Study	Living in Hong Kong	
High school or higher (% yes)	74.2	64.0	0.12	74.4	NA^c^	NA
Age at menarche (years)	14.6 (1.8)	15.0 (2.0)	0.03	12.8 (1.9)	15.1 (2.3)^d^	<0.001
Age at first live birth (years)	25.2 (3.6)	24.0 (4.0)	0.001	26.9 (3.9)	NA^c^	NA
Number of live births	2.2 (1.1)	2.3 (1.0)	0.30	2.1 (1.4)	2.0 (2.0)^e^	0.59
Age at natural menopause (years)	50.0 (3.4)	48.0 (5.0)	<0.001	50.0 (3.2)	49.4 (3.4)^c^	0.08
BMI (kg/m^2^)	23.2 (3.0)	24.2 (3.6)	0.002	22.6 (3.2)	22.2 (3.8)^e ^to 23.6 (3.4)^d^	0.58^f^

^a ^*P *values were based on chi-square significance test for educational attainment and t-tests for the rest of the variables.

^b ^Data obtained from reference 10.

^c ^Data is not available.

^d ^Data obtained from reference 9

^e ^Data obtained from reference 8

^f ^Weighted mean was used for postmenopausal Chinese women living in Hong Kong when making the comparison.

### Estimated five-year absolute risk for breast cancer

**Figure 1:**
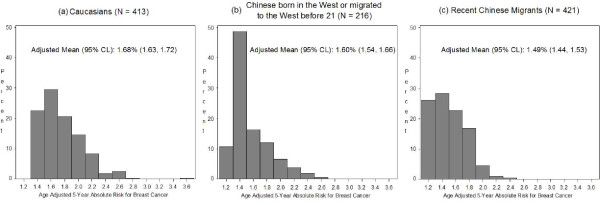
Estimated five-year absolute risk for breast cancer in Caucasians. Application of the Gail Model to estimate five-year absolute risk for breast cancer in Caucasians with **(a) **risk factor profile of Caucasians **(b) **risk factor profile of Western born Chinese and early Chinese migrants **(c) **risk factor profile of recent Chinese migrants. *P *values were based on ANCOVA, adjusting for age. Log Transformation was used. *P *values: Ethnicity effect: <0.001; Caucasians vs. Chinese born in the West or early Chinese migrants: 0.35; Caucasians vs. recent Chinese migrants: <0.001; and Chinese born in the West or early Chinese migrants vs. recent Chinese migrants: 0.005.

Figure 1 shows the age-adjusted five-year absolute risk for breast cancer estimated using the Gail model. The estimated five-year absolute risk for Caucasians was 1.68. If Caucasians were to have the risk factor profile of Western born Chinese and early Chinese migrants or that of recent Chinese migrants for the variables in the Gail Model, their five-year risk estimates would decrease by 5% to 1.60 and 11% to 1.49 respectively.

## Discussion

To our knowledge, this is the largest study to date on Chinese migrants defined by place of origin and time of migration. We collected detailed information on factors that may influence breast cancer risk in three groups of postmenopausal women who are members of populations at substantially different risk of breast cancer. Although our recent Chinese migrants may be slightly more westernized than Chinese women living in urban China, there is a two-fold difference in breast cancer incidence between Canada and urban areas in China [1], and a 37% lower breast cancer mortality in Canadian women who were born in China compared to those born in Canada[3]. Further, breast cancer incidence in migrants from Asia to the United States has been shown to remain closer to the level of the country of origin for a decade after migration [2].

Despite the one-child policy, number of live births was comparable in our recent Chinese migrants and postmenopausal women living in urban China. This is likely because our recent Chinese migrants, with a median year of birth of 1945 and age at first live birth attained in 1971 at a median of 26 years, were not as affected by the one-child policy, which was implemented in 1979. Nevertheless, the one-child policy is a significant limitation of using Chinese migrants as a proxy for Chinese women in a younger study population.

Our results show that levels of exposure to most of the risk factors examined were highest in Caucasians, intermediate in Western born Chinese and early Chinese migrants, and lowest in recent Chinese migrants. Among recent Chinese migrants, those from Hong Kong had the highest exposure to all of the risk factors examined, except for height. Northern Chinese are taller than southern Chinese [11], and migrants from Mainland China were both northern and southern Chinese, while migrants from Hong Kong were primarily southern Chinese. Leg length reflects childhood nutrition and other influences on growth [12], and explained most of the difference in height between the groups.

Some risk factors were more strongly associated with migration history than others. Compared to recent Chinese migrants, Western born Chinese and early Chinese migrants had slightly higher use of hormone replacement therapy and significantly higher oral contraceptive use. This is probably because use of alternative therapies, such as traditional herbal remedies, is common in Asians living in Western countries, regardless of migration status, for relief of menopausal symptoms [13], and these therapies cannot serve as an alternative to oral contraceptives for birth control. Although alcohol and tobacco use increased with increasing time of residence in the West, use was much lower in both Chinese groups than in Caucasians, suggesting maintenance of the tradition in Chinese culture that discourages women from smoking and drinking [14,15].

Western born Chinese and early Chinese migrants had significantly lower BMI but similar skinfold thicknesses compared to Caucasians, and had slightly higher BMI and much larger skinfold thicknesses compared to recent Chinese migrants, possibly as a result of differences in body composition. Since adipose tissue is the sole source of endogenous estrogens in postmenopausal women, these results suggest that acculturation may increase breast cancer risk in postmenopausal women in part by increasing body fat and thus exposure to estrogens, which is associated with breast cancer risk[16].

Survey data showed that prevalence of breastfeeding was lower in Hong Kong than in Western countries and Mainland China [17]. This is seen in our data that recent Chinese migrants from Hong Kong had the lowest breastfeeding rates among the study groups. Another risk factor that recent Chinese migrants had higher exposure to was more frequent radiation exposure to the chest area, which is likely the result of efforts to control the high prevalence of tuberculosis in China by population screening with chest x-rays [18,19].

Compared to the other groups, recent Chinese migrants less frequently reported a history of breast cancer in first-degree relatives, reflecting the lower incidence of breast cancer in China [1]. It is unlikely to be due to physical barriers to communication with family living in the East, as modern communication technology and frequent visits enable Chinese migrants to keep informed about their families in the homelands [20].

Recent Chinese migrants had the lowest breast biopsy rates among the groups. This may be attributed in part to mammography screening being more common in the West than in China [21], rather than to difference in breast disease rates alone.

To date, only two other migrant studies have examined the distribution of breast cancer risk factors in Asian women according to acculturation. Our results are similar to those of a cross-sectional study in 212 foreign-born American-Chinese women by Tseng el al [22], which showed that more acculturated women had lower age at menarche, fewer children, were more likely to have used oral contraceptives, and that more acculturated postmenopausal women were more likely to have used hormone replacement therapy. However, our results differ from Tseng et al's [22] findings that more acculturated women had higher age at first live birth. The later age at first live birth observed in our recent Chinese migrants may be a result of their exposure to the Cultural Revolution in Mainland China, when the fertility rate dropped [23]. Also, starting in the 1960s, significant improvements in education resulted in increased employment and postponement of marriage and childbearing in Hong Kong women [24,25]. Differences in study populations may explain differences in our results as Tseng et al [22] included women born in Southeast Asia and Taiwan as well as Hong Kong and Mainland China.

A case-control study in Asian American women (597 cases and 966 controls) by Ziegler et al [2] showed that breast cancer risk was higher in Asian American women born in the West than those born in the East, and that Asian born women who were long-term residents in the West had higher breast cancer risk than more recent migrants. In the same study population, Wu et al[26] showed that Asian American women born in the West had later age at both menarche and at first live birth than Asian migrants, which is in agreement with our findings.

By applying the Gail Model [5,6] to the study groups, we found that if Caucasians had the risk factor profile of recent Chinese migrants for the variables in the Gail Model, their risk, which is at least twice that of the Chinese migrants, would decrease by an estimated of only 11%, suggesting that there likely are other risk factors that also contribute to the difference in breast cancer incidence between Canada and China. In this context, it is relevant to note that a recent study suggested that modest reductions in postmenopausal hormone use and alcohol consumption, and weight maintenance, may prevent approximately 10% of the 2.5 million breast cancer cases predicted in China by 2021 among Chinese women who were 35 to 49 years old in 2001 [27], and that postmenopausal hormone therapy, alcohol consumption, and weight are not included in the Gail Model.

A limitation of the present study is the small number of Western born Chinese and early Chinese migrants, most of whom in the populations from which we recruited were premenopausal and not eligible for this study. Recruitment of subjects through mammography clinics is another potential limitation of this study, as women who participate in breast screening programmes may be healthier and more health conscious, than the general population. Also, mammography use is underutilized among Chinese-Canadian women [28], reducing the generalizability of our results.

## Conclusions

Our results suggest that in addition to the risk factors in the Gail Model, there likely are other factors that also contribute to the large difference in breast cancer risk between Canada and China. Our dietary acculturation results showed that the study groups had different eating patterns. In separate papers, we will compare dietary intakes and physical activity between Chinese and Caucasian women from this study.

## Abbreviations

ANCOVA: analysis of covariance; ANOVA: analysis of variance; OBSP: Ontario Breast Screening Programme; SMPBC: Screening Mammography Programme of British Columbia.

## Competing interests

The authors declare that they have no competing interests.

## Authors' contributions

CT, LJM, GH, SM, and NFB were responsible for the study design; CT coordinated the study, performed data analyses, and drafted the manuscript; and CT, LJM, AJH, SM, and NFB were responsible for data interpretation. CT, LJM, GH, AJH, SM, and NFB edited the manuscript. All authors read and approved the final manuscript.
